# Haplotype and minimum-chimerism consensus determination using short sequence data

**DOI:** 10.1186/1471-2164-13-S2-S4

**Published:** 2012-04-12

**Authors:** Shawn T O'Neil, Scott J Emrich

**Affiliations:** 1 Department of Computer Science and Engineering, University of Notre Dame, Notre Dame, IN 46556, USA

## Abstract

**Background:**

Assembling haplotypes given sequence data derived from a single individual is a well studied problem, but only recently has haplotype assembly been considered for population-sampled data. We discuss a software tool called Hapler, which is designed specifically for low-diversity, low-coverage data such as ecological samples derived from natural populations. Because such data may contain error as well as ambiguous haplotype information, we developed methods that increase confidence in these assemblies. Hapler also reconstructs full consensus sequences while minimizing and identifying possible chimeric points.

**Results:**

Experiments on simulated data indicate that Hapler is effective at assembling haplotypes from gene-sized alignments of short reads. Further, in our tests Hapler-generated consensus sequences are less chimeric than the alternative consensus approaches of majority vote and viral quasispecies estimation regardless of error rate, read length, or population haplotype bias.

**Conclusions:**

The analysis of genetically diverse sequence data is increasingly common, particularly in the field of ecoinformatics where transcriptome sequencing of natural populations is a cost effective alternative to genome sequencing. For such studies, it is important to consider and identify haplotype diversity. Hapler provides robust haplotype information and identifies possible phasing errors in consensus sequences, providing valuable information for population studies and downstream usage of resulting assemblies.

## Background

The assembly and analysis of short-read sequence data presents a number of well known challenges including error correction, correct determination of repetitive regions, and accurate identification of genetic variation such as single nucleotide polymorphisms (SNPs) and insertions/deletions (indels). The end result of an assembly is a set of consensus sequences that ideally matches genetic sequence found on naturally occurring chromosomes. When input reads are all sourced from highly inbred individuals (e.g., from clonal lines of *Drosophila*), this is easy to ensure: any variation should result from sequencing error, and the popular "majority vote" mechanism will create a correct consensus [1].

When reads are sourced from non-inbred individuals, the majority vote mechanism reduces to an uninformed parsimony approach; we assume the existence of a "most frequent" consensus and rely on coverage depth to identify it. Unfortunately, this approach disregards the extant sequence diversity, and does not identify possible errors in the consensus assembly caused by it. Thus, proper analysis of diverse data should focus on the assembly or reassembly of haplotypes--consensus sequences that match to at least one of the diverse set of chromosomes in the sample.

Reconstructing haplotypes from low cost, low fidelity data sources has been an active area of research for over 20 years [2], but not always in the context of sequence assembly. For example, genotype (SNP-chip) data sourced from many diploid individuals of a population or lineage provides alleles present in each individual, but does not associate co-occurring alleles across loci and requires statistical methods to infer haplotypes [3].

Haplotypes can also be reconstructed given an assembly (or alignment against a reference) of the polymorphic reads. Because reads may span multiple variant loci, each read may contain a small amount of haplotype information. When the number of haplotypes is known to be two, this is known as the *single-individual haplotype assembly problem*.

Here, we are interested in a recent variant of the problem: assembling haplotypes from read data where the true number of source haplotypes is unknown, which we call the *population haplotype assembly problem*. For the related problem of viral quasispecies estimation, Eriksson et al. and Zagondi et al. previously formulated a graph-theoretic solution called ShoRAH [4,5], which was applied to high diversity, high coverage data (e.g., HIV data representing 6.8% pairwise genetic divergence sequenced to 2100X [5]). This application benefits from high diversity, because overlapping individual reads are likely to share informative SNPs, and errors can be more clearly identified because of high coverage.

Many recent biological studies, however, have sequenced genetic data from pools of higher-order individuals. For example, population-level *de novo *transcriptome assemblies provide a low cost alternative to whole genome sequencing for ecologically important non-model species [6]. Such populations represent less genetic diversity (6 to 20 SNPs per kilobase) and are sequenced to shallower depths (6-23X) [7-11]. For haplotyping, these properties result in difficulties that we address here:

• Less concrete haplotype information is available.

• Sequencing errors are more difficult to identify.

This paper expands on Hapler [12], a tool for assembling reliable haplotypes given a preliminary alignment of genetically diverse reads. Here, we describe and discuss the methods used by Hapler in detail, including a new feature that reconstructs full consensus sequences. This reconstruction identifies and minimizes possible chimeric crossover points (points at which different haplotypes may be contributing to the consensus due to lack of phasing information) while maximizing the read coverage, effectively recovering the parsimony principle of majority vote. Because the length of discernible haplotype regions is usually limited by read length, we focus on gene-sized alignments as would be found in transcriptome studies. The core of the graph theoretic formulation of Eriksson et al. relies on a maximum unweighted matching of a bipartite representation of the alignment. Our formulation builds on this and incorporates 1) *in-situ *isolation of reads likely containing errors, via a weighted matching of a related representation, and 2) parameter adjustable "quality" constraints, reducing the probability of reconstructing chimeric haplotypes (see Methods). This is accomplished by exploiting the mechanism of the Hungarian weighted matching method, sampling from the space of possible haplotypings and retaining only commonalities; by default, this parameter is kept high to ensure high-quality output.

We test Hapler by simulating sequencing, alignment, haplotype assembly and consensus reconstruction for population-sourced data representing diversities of 1.2% and 4.4%. These simulations indicate that the novel methods employed by Hapler effectively assemble correct haplotype regions, and that the quality of results produced will scale with the quality of future input data: as datasets grow to contain longer reads and fewer sequencing errors, more correct and complete haplotypes will result.

Finally, we find that the consensus sequences produced include less chimerism than related approaches of quasispecies estimation and majority vote.

### Related work

Combinatorial approaches for the single-individual haplotype assembly problem abound (e.g. [13-16]). In these approaches, certain columns in the alignment *M *are identified as SNP columns. Based on these, a *conflict graph G_M _*is created wherein each node represents one read, and nodes in *G_M _*are connected if their reads *conflict*, i.e., overlap at some SNP column and disagree in the allele. Some SNP columns may contain *gaps*, positions where the allele is unknown. In this paper, we distinguish deletion alleles ('*-*') that cause conflicts from gap positions ('*~*'), which do not. For example, paired-end reads and newer strobe reads can be viewed as long sequences containing one or more sections of contiguous gaps that do not contain discernible SNPs.

In the single individual haplotype assembly problem, the conflict graph will necessarily be bipartite in the absence of sequencing errors. In this case, computing the bipartition reconstructs haplotypes. When sequencing errors are present, the graph may not be bipartite. Various optimizations exist for inducing bipartiteness such as Minimum SNP Removal (MSR) and Minimum Fragment Removal (MFR), though these are NP-Hard unless the reads are all gapless [17]. Other models are NP-Hard even in the gapless case [18]. Because of similar theoretical constraints in Hapler, all results presented here assume gapless reads (see Conclusion, Methods).

Recently, the MFR problem has been extended to when the number of known haplotypes is increased from two to a known constant *k *[19]. Non-graph theoretic approaches also exist for the single individual haplotype assembly problem (e.g [20,21]). The related problem of determining haplotypes from unphased genotype "SNP-chip" data of several diploid individuals has also received extensive attention; see Salem et al. for a comprehensive review [3].

**Figure 1:**
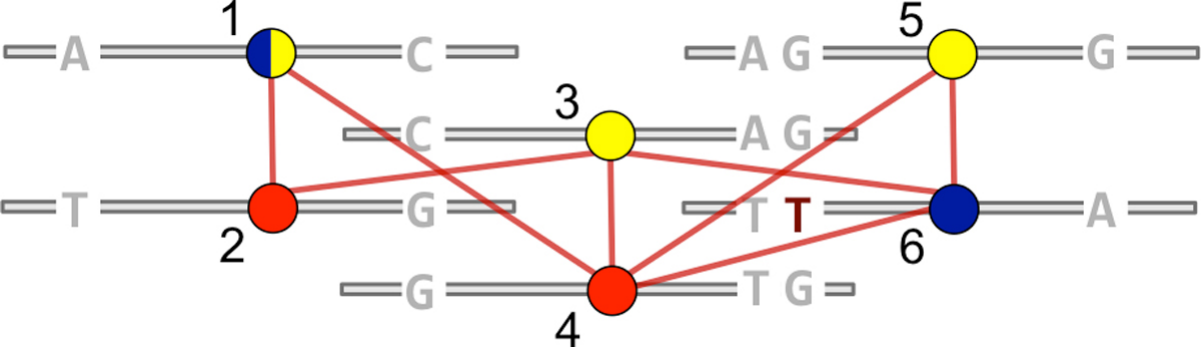
**Example conflict graph *G_M _*overlayed on a multiple alignment, with alleles shown only for variant loci**. These reads likely represent two haplotypes, with a sequencing error in read number 6. Hapler considers that there are many possible colorings of conflict graphs (e.g., dark and light are both proper colorings for read number 1), each representing a different reconstruction consistent with the data.

In our formulation of the population haplotype assembly problem, the goal is not to bipartition *G_M_*, but rather to minimally color it--or, equivalently, minimally clique cover the complement "compatibility" graph $G_{M}^{\prime }$--assuming sequencing errors have been addressed (Figure 1). In the context of the quasispecies estimation problem, Eriksson et al. observed that with gapless data the set of irredundant reads and their conflict information induce a partial order. Thus, Dilworth's theorem applies, and it is sufficient to find a chain decomposition of a transitive orientation, $\vec{{G^{\prime }}_{M}}$, of the compatibility graph. Such a chain decomposition is found via a maximum unweighted matching in a bipartite graph representation of the "reachability" relationships in $\vec{{G^{\prime }}_{M}}$. Recently, the method of Eriksson et al. has been improved upon by Astrovskaya et al., who incorporated probabilistic weights [22]. Both of these methods attempt to reconstruct full end-to-end haplotype consensus sequences by relying on suffcient per-haplotype coverage [4,5].

Reconstructing consensus sequences with a focus on identifying and minimizing chimerism has been minimally studied. For single individual haplotype assembly, the number of possible crossover points is fully determined by the number of connected components with at least two nodes in the bipartite conflict graph [20]. The Celera Assembler incorporated a window-based phasing heuristic, reporting a number of "variant regions" for each consensus [23].

## Results and discussion

We previously tested Hapler's haplotype assembly abilities on a low diversity dataset, including a single outgroup haplotype for higher diversity tests [12]. These initial results suggested that Hapler can effectively assemble haplotype regions, however the inclusion of only a single outgroup sequence limited the generality of the conclusions. Here, we test both Hapler's haplotyping and consensus reconstruction abilities on data sourced from a 600 bp barcoding region of the COI gene of *Melitaea cinxia*, a well-studied butterfly. Of the known haplotypes for this gene, we chose eight from two distinct clades (Supplementary Figure 1, Additional File 1[24]). The first, "Clade 1," consists of four haplotypes containing 7 SNPs for a total diversity of 1.16%, just below that estimated for the transcriptome as a whole (1.2%, [8]). This clade represents our "low diversity" test data. The second clade also consists of four haplotypes, and contains 10 SNPs. When combined, these two clades contain 24 SNPs, for a total diversity of 4.0%. These combined clades represent our "high diversity" data.

Unless otherwise specified, we simulated sequencing and perfect assembly by randomly selecting subsequences of haplotypes of a specific length. For the low diversity dataset, we sequenced each of the four haplotypes to 6X coverage to achieve total coverage similar to recent transcriptome sequencing projects (23X in [11]). For the high diversity dataset, each of the eight haplotypes was sequenced to 3X coverage. Because we are interested in testing up to Sanger size read lengths on full gene transcripts, each COI barcoding variant was quadrupled in length by concatenating the COI variant, the variant with T's and C's switched, the variant with A's and G's switched, and the variant with A's and T's switched. This process retains the same level of diversity and allows for comparisons to other tools that map reads to a reference. This process did not introduce inter-SNP distances longer than those present in the original COI barcoding variants.

If a sequencing error *e *is specified, each base of each read is mutated to one of the remaining three bases (uniformly chosen) with probability *e*. Unless otherwise specified, default parameters were used for Hapler ("Binomial" SNP calling with default parameters, minimum of 20 coloring repetitions, see Methods).

### Read length and diversity

We first tested Hapler's ability to assemble correct haplotypes by varying simulated read lengths without sequencing error and assuming perfect SNP calling. For each read length, we assembled haplotypes over 5 trials of random simulated sequencing. All haplotype regions reported here exclude the "universal" haplotype, which is composed of reads that are common to all complete haplotypes (see Methods). For all tests, we call a haplotype assembly correct if it is an *exact *subsequence of some original COI sequence, otherwise it is incorrect.

**Figure 2:**
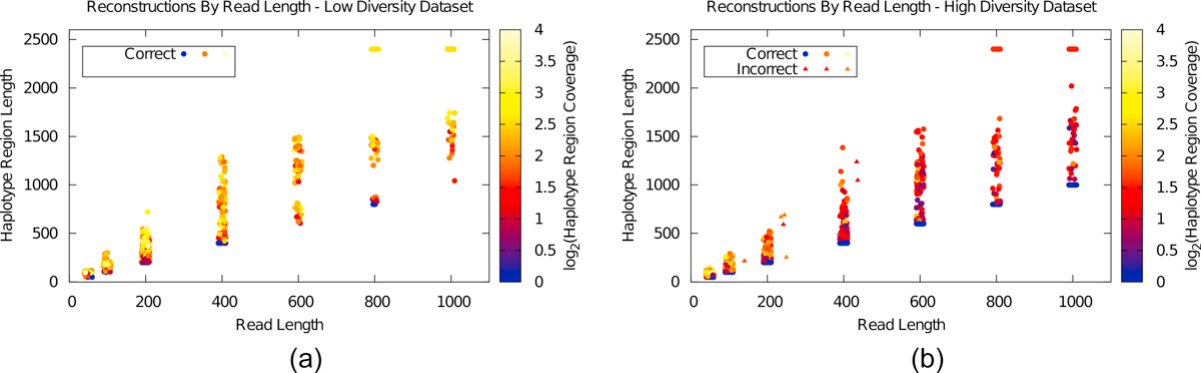
**Haplotype assemblies of the low and high diversity datasets to 24X total coverage, varying read length, with 0% error rate**. Correct haplotype assemblies (see text) are represented with circles; erroneous assemblies are shown as triangles and offset for legibility. For these five trials, chimerism is rare and longer read lengths provide longer haplotype assemblies as expected.

Figure 2(a) shows the results for the low diversity data: each point represents an assembled haplotype region. The vertical axis shows haplotype assembly length (see Methods for details) while color indicates coverage, determined by non-redundant and accompanying redundant reads assigned to that haplotype. Horizontal-axis jitter has been added for readability. Similar results are shown in Figure 2(b) for the high-diversity dataset.

Results for both datasets are largely similar: in both cases, longer sequences result in longer haplotypes, and longer haplotypes tend to have higher coverage. In both cases, we see improvements between 200 bp and 400 bp reads. This is unsurprising, as all extended COI variants share identical regions with other variants of size ≈ 250 bp. Lack of *>*250 bp reads prevents phasing across these regions, which Hapler correctly infers. For the low diversity dataset, all reconstructed haplotypes were correct; for the high diversity dataset, of the 2,766 assembled sequences only seven were incorrect chimeras, shown offset for readability in Figure 2(b).

### Error rate

**Figure 3:**
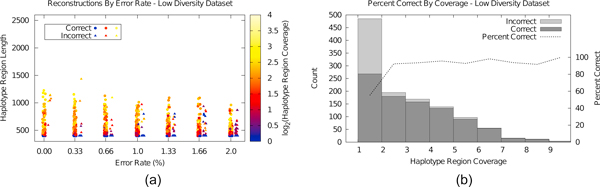
**Haplotype assemblies varying error rate for the low diversity dataset over five trials of sequencing to 24X total coverage**. Figure 3(b) aggregates all results of 3(a) binned by coverage, identifying counts and percents of correct versus incorrect assemblies. Even with high rates of sequencing error, high coverage indicates a likely correct assembly.

Next, we tested Hapler's performance with sequencing errors. For these tests, we fixed read lengths to 400 bp (representative of 454 FLX Titanium technology). In order to show the results for haplotype assembly in the general case, for each sequencing error rate *e *in Figure 3(a), we show the haplotypes assembled over 5 trials.

As the error rate increases, the average length of correct haplotypes decreases, while the number of incorrect haplotypes increases. Length of incorrect haplotypes is comparable to correct haplotypes, particularly for high error rates. However, when error rate is high the average coverage of incorrect haplotypes is low, indicating that Hapler successfully isolates sequencing errors as anomalous, rare haplotypes with little support.

Figure 3(b) shows counts of all correct and incorrect haplotype assemblies of Figure 3(a) binned by coverage, as well as the percentage correct for each bin. This figure indicates that average cutoff can be an effective metric for determining whether haplotypes represent reality--at 2X or higher coverage, nearly all assembled haplotypes are correct as the within-haplotype majority vote mechanism (see Methods) is able to filter out sequencing errors. Results for the high diversity dataset were similar, though haplotype assembly lengths suffered in comparison, particularly at high error rates where true SNPs are difficult to distinguish from rare alleles (Supplementary Figures 2(a) and 2(b), Additional File 1). When coverage is doubled, results are improved at the highest error rates (Supplementary Figures 2(c) and 2(d), Additional File 1). For this test, we also evaluated the full length consensus sequences produced by Hapler, the majority vote, and the most populous quasispecies estimated by ViSpA [22]. We emphasize that while ViSpA can account for many haplotypes, it is designed for much higher diversity and sequencing depths for viral quasispecies applications; here we use parameters *n *= 1 and *t *= 80 as appropriate for lower-diversity datasets [22]. Although a wide variety of software exists for the single individual haplotype assembly problem, these solutions assume two haplotypes are to be constructed, and thus would be inappropriate for comparison to ViSpA and Hapler for data representing many haplotypes.

We evaluated each of these consensus generation techniques by computing the minimum number of crossovers (pairs of SNP loci which must be supported by different haplotypes) necessary to reconstruct the consensus amongst 1) original input haplotypes, representing "true" crossovers, and 2) Hapler assembled haplotypes, representing "estimated" crossovers; in both cases, sequencing errors included in consensus sequences at SNP positions also incur crossovers (see Methods). For chimerism analyses, each data point is an average of 50 sequencings/assemblies/consensus reconstructions.

**Figure 4:**
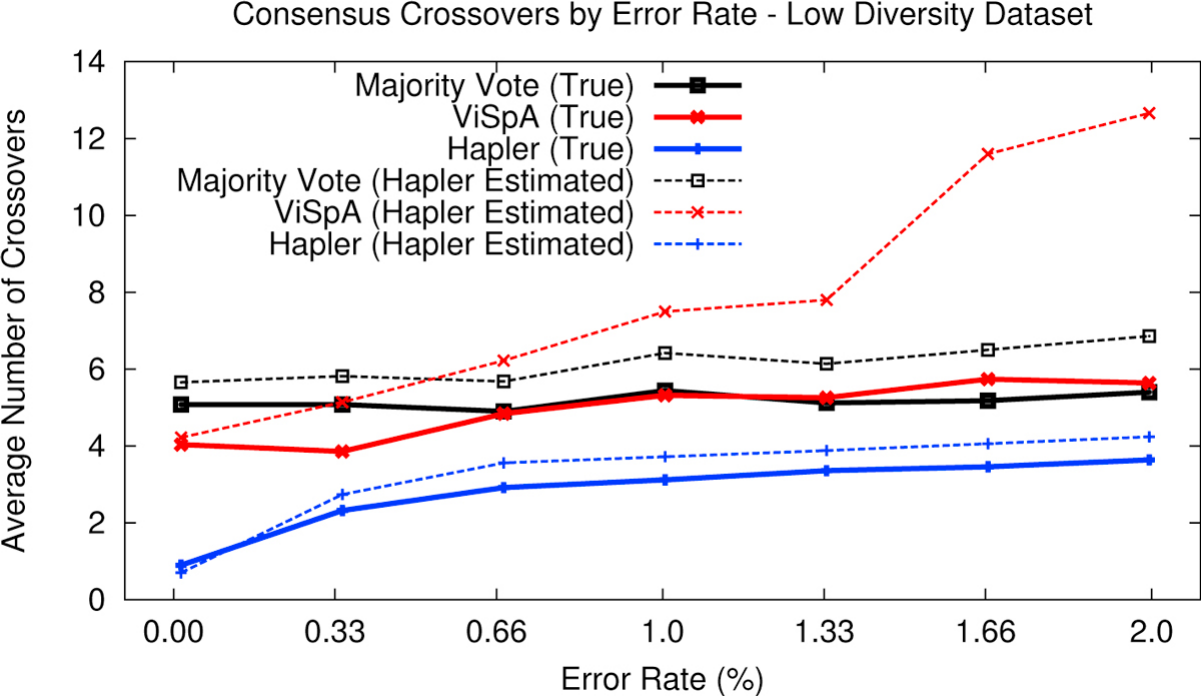
**Chimerism analysis varying error rate**. True crossover numbers indicate the minimum number of crossovers through sequenced haplotypes needed to reconstruct the consensus. Hapler estimated crossover numbers indicate the minimum number of crossovers through Hapler-assembled haplotypes needed to reconstruct the consensus. Each datapoint represents an average of 50 measurements.

Figure 4 shows the chimerism analysis varying error rate. At low error rates, the Hapler consensus contains between 0.5 and two true chimerisms on average, increasing to just over three at higher error rates. The majority vote holds steady at ≈ 5 crossover points on average, regardless of error rate. ViSpA significantly improves on the results of ShoRAH (consistently incurring ≈ 12 crossovers, not shown) and improves on the majority vote, particularly at low error rates.

Hapler's estimate of the number of crossover points trends between one and two above the true number for its own and the majority vote consensus. At high error rates, the estimated number of crossovers for the ViSpA consensus diverges from the true number faster than for the other two. For the high diversity dataset (Supplementary Figure 3, Additional File 1), the true number of crossovers for Hapler only increases by approximately one, whereas the number for the majority vote suffers significantly incurring over 10 crossovers on average.

### Random repetitions

**Figure 5:**
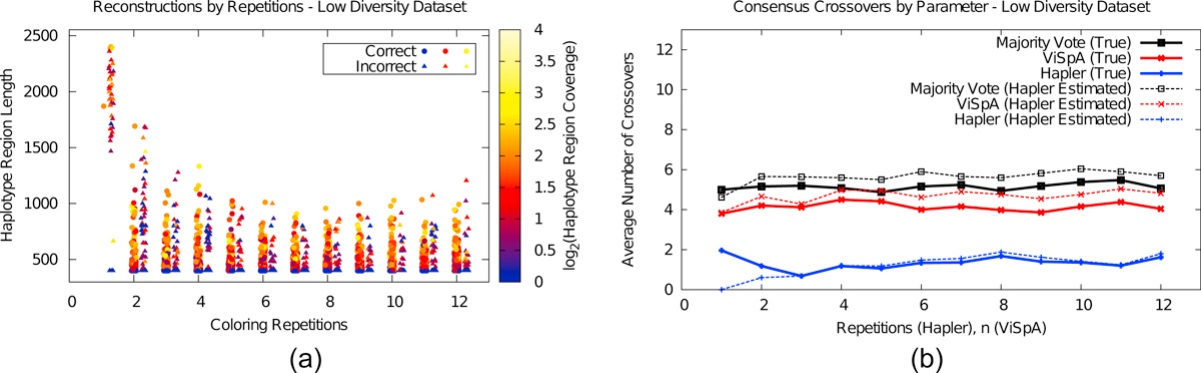
**Haplotype assemblies and consensus chimerism analysis varying coloring repetitions (for Hapler) and *n *(for ViSpA) for the low diversity dataset**. Figure 5(a) shows that haplotype assemblies are shorter but less likely to be chimeric as the number of repetitions increases, while 5(b) shows that when full length consensus sequences are reconstructed, true rates of chimerism are low even when few repetitions are used.

Because many possible haplotypings may exist, we randomly repeat the colorings that produce haplotype assemblies and only infer information common to all of them (see Methods). By default, Hapler runs a minimum of 20 such repetitions, and continues until the number of haplotypes assembled has stabilized for the previous 50% of repetitions. For Figure 5(a), we use artificially low numbers of repetitions on the low diversity dataset; read length was held at 400 bp and the error rate was held at 0.01, and we again show haplotype assemblies produced over 5 trials of sequencing.

With only a single minimum coloring most haplotype assemblies, while long, are incorrect chimeras. As the number of repetitions increases, correct haplotype inferences quickly begin to outnumber incorrect ones and the lengths of both correct and incorrect haplotypes drop. We quickly see an asymptotic behavior: correct haplotypes reach a minimum length and incorrect haplotypes reach a small average coverage. Results were similar for the high diversity dataset (Supplementary Figure 4(a), Additional File 1). Figure 5(b) shows the chimerism analysis for consensus reconstructions. While we varied the number of repetitions used by Hapler from 1 to 12, we also varied the *n *parameter used by ViSpA from 1 to 12. When the number of repetitions is very small, we see Hapler for the first time consistently underestimating the number of crossovers. In this case, although the number of true crossovers is steady for Hapler's consensus sequences at approximately two, Hapler estimates zero crossovers because of its assembly of long (but actually chimeric) haplotypes. For both the low and high diversity datasets, the estimated chimerism approximates or exceeds the true chimerism after four colorings (Figure 5(b), Supplementary Figure 4(b), Additional File 1).

### Unequal haplotype representation

**Figure 6:**
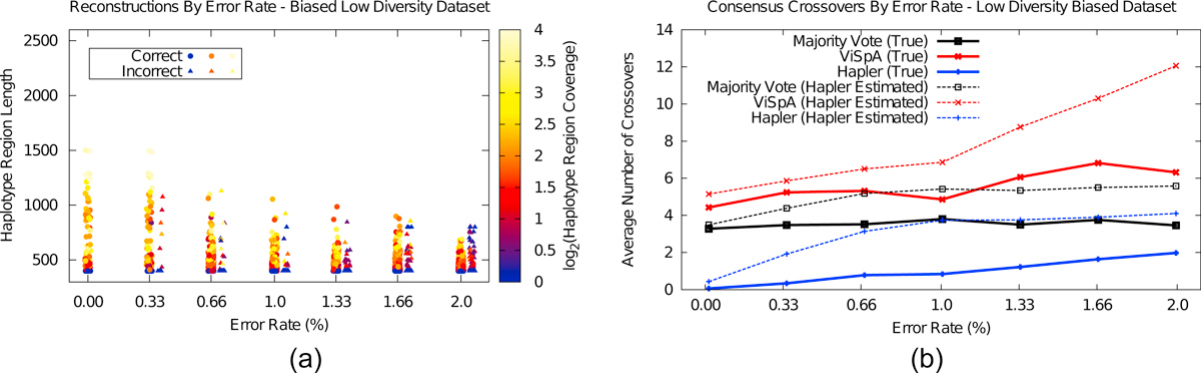
**Haplotype assemblies and chimerism analysis for the low diversity dataset with a single over-represented haplotype**. These results indicate that high error rates reduce the length of haplotype assemblies compared to equal haplotype representation, however true chimerism is generally lower in reconstructed consensus sequences.

The situation tested thus far--equal representation of several haplotypes--represents a difficult scenario for the majority vote mechanism, which is expected to perform better when a single haplotype is more prevalent. In this section, we modified the low diversity dataset such that one haplotype is represented three times in the "population" while the other three haplotypes are represented once. We simulated sequencing of these to 4X coverage each (12X for the most populous haplotype, 4X each for the remaining three) keeping the read length at 400 bp and varying error rate. Figure 6(a) shows the haplotype region assembly results over 5 trials of sequencing; these are similar to those of Figure 3(a), though at high error rates assembled haplotypes are shorter.

Figure 6(b) shows the chimerism results for consensus sequences. The majority vote and Hapler are able to reduce chimerism overall in this test, while ViSpA shows improvement at low error rates. Even with a single haplotype representing 50% of the population, the majority vote consistently contains approximately three crossovers while Hapler significantly improves on this, particularly for low error rates. The continued poor results for the majority vote may seem surprising, however, there are four loci in the expanded COI haplotypes where the most populous haplotype differs from all others. Majority vote requires not only a most populous haplotype, but a true majority haplotype. This is not always guaranteed, particularly in populations with high gene flow (see, e.g., [25]).

### Runtime

Hapler's robustness comes at a computational cost: repeated weighted matching dominates the runtime at *O *(*rn*^3^), where *r *is the number of repetitions and *n *is the number of irredundant reads in the largest haplotype block (see Methods). Even when the largest haplotype block is small, reconstruction of the minimum chimerism consensus runs in *O *(*mk*^2^) time and space, where *m *is the number of called SNPs and *k *is the number of haplotype regions.

Table 1 shows runtimes, simulating sequencing of the low diversity dataset with 400 bp reads and a high error rate of 5% to various depths. Using the "Simple" SNP caller ensured that each read would be considered as an irredundant read that would also become an individual haplotype assembly (# reads = *n *= *k*). These tests were run on a dual core 2.2 GHz desktop with 8 GB of RAM, though all tests used only a single core and less than 200 MB of resident memory. Hapler used 100 repetitions.

**Table 1:** Runtimes

Coverage	# SNPs	# Reads	Runtime
5X	1482	120	15 secs
10X	1970	240	61 secs
15X	2186	360	155 secs
20X	2280	480	430 secs

Runtimes, simulating sequencing of the low diversity dataset with high error rate and "Simple" SNP caller (so that each read is considered as a unique haplotype).

As expected, runtimes grow super-linearly with the number of reads (though perhaps not as quickly as the worst case *n*^3 ^factor would suggest). For comparison to the Table 1, we consider an example 114 kilobase contig of the population-sourced *Anopheles gambiae *genome assembly (Lawniczak et al., [26]) comprised of 984 reads containing 879 non-redundant reads with the largest of 12 haplotype blocks containing 481. For this contig, Hapler called 2,845 SNPs (using the "Simple Strict" SNP caller, see Methods) and reconstructed a consensus with 146 estimated crossovers in 12 minutes 38 seconds. All individual Hapler runs contributing to Figures 2 through 6 ran in under 12 seconds.

## Conclusions

Until recently, the analysis of genetically diverse short read data has focused on identifying single-locus polymorphisms such as SNPs (and, using mate-pair data, longer polymorphisms such as inversions and long indels [27]). However, haplotypic information is often more useful than locus-specific information [28,29]. Here, we described a new software tool called Hapler designed to accurately assemble haplotypes from low diversity, low coverage data. Assemblies can be improved by minimizing the number of haplotypes supported by the data while maximizing the number with minimal support, and sampling from the many possible consistent haplotypings to infer only robust haplotype regions (see Methods).

The theoretical foundations of Hapler require that sequences be gapless. In support of this requirement, initial experiments that incorporated mate-pair information only produced few incorrect haplotypes, with many internal errors. For this reason Hapler currently does not incorporate mate-pair information. Hapler's added robustness does come at a computational cost, but experimental runtime analysis indicates that runtime will nevertheless be adequate for single alignments up to 100 kilobases in length, though low levels of phasing information are likely to prevent useful results over regions of this size. Runtimes also suggest that Hapler will be useful in practice for whole-transcriptome assemblies, where contig alignments are on the order of a few kilobases in length.

Although low levels of phasing information may prevent assembly of many long haplotype regions in some applications, Hapler can use the few long and highly covered regions to produce a full consensus minimizing possible chimerism. In this regard, Hapler performs well compared to the majority vote and quasispecies estimation tools (using the most frequent quasispecies estimate as the consensus). Perhaps most telling, of the 350 consensus sequences produced by Hapler over various error rates for Figure 4, 20 were exact matches to some sequenced haplotype, whereas the other approaches produced no exact matches. Results were similar when varying parameters (Hapler: 185, ViSpA: 2, majority vote: 0) and in the unequal representation test (Hapler: 208, ViSpA: 3, majority vote: 4). We emphasize that quasispecies estimation tools are designed for datasets of much higher coverage and diversity, and thus optimize criteria and utilize parameters specific to those applications.

Hapler can estimate the chimerism of any consensus against its own haplotype region assemblies. This estimate will naturally overestimate the true chimerism (due to Hapler's conservative methods); however, in most tests Hapler's estimated chimerism for the approaches tested preserved the ranking of true chimerism. The only exception we found was when error rates were high (Figure 4), and Hapler estimated ViSpA as having a higher chimerism than the majority vote, when in fact ViSpA performed similarly to or slightly better than the majority vote.

Finally, Hapler not only minimizes chimerism in its consensus reconstructions, it also identifies where possible chimeric points might occur. This is useful information, for example, when designing microarray probes or PCR primers that may fail if they include chimeric sequence.

### Software availability

Hapler is packaged as a single Java.jar file, and has been tested with Java 1.6, requiring no other dependencies. It is available at http://www.nd.edu/~biocmp/hapler and is released under an LGPL license. Hapler is also easy to use: to reconstruct haplotypes for a list of contig IDs in an Amos bank, one can simply use the Amos tools in conjunction with Hapler: bank2contig -E eIDList.txt amosBank | java - jar Hapler.jar --input -.

## Methods

Hapler takes as input read alignments either in TIGR [30] or SAM [31] format, as well as an optional list of positions to consider as variant loci. A polynomial time solution requires that reads do not contain gaps, thus mate-pair information is currently ignored. For each multiple alignment, Hapler executes the following steps:

1. (Optional) SNP calling.

2. Masking of redundant reads.

3. Determination of haplotype blocks from connected components of *G_M_*, and determination of the "universal" haplotype.

4. For each haplotype block *M *':

(a) Determining the transitive compatibility graph $\vec{{G^{\prime }}_{M^{\prime }}}$.

(b) Minimum coloring of $G_{M^{\prime }}^{\prime }$, maximizing the number of single-node colors.

(c) Randomly repeating step b), identifying commonalities in colorings, and assembling haplotype regions.

5. Reconstructing an overall consensus, minimizing possible crossover points while maximizing SNP allele coverage.

6. If given a consensus sequence to evaluate, computing the minimum number of crossovers to support it with haplotype regions. If also given a set of "true" haplotypes, computing the minimum number of crossovers through these to support the given and reconstructed consensus sequences.

As the sections below discuss, it is primarily steps 3), 4b), and 4c) that improve haplotype assembly quality.

### SNP calling

Although step 4b) is designed to isolate reads that likely contain errors so they can be ignored (see below), many sequencing technologies induce frequent errors that are easy to identify. For example, 454 data is plagued by indel errors adjacent to homopolymer runs [32]. Hapler currently incorporates a number of methods for identifying which variant loci should be used in haplotyping:

• Simple: Any variant locus regardless of allele frequency is treated as a SNP locus.

• Simple Strict (default): Any variant locus where a minority allele is present at least twice or is covered by less than 10 reads is treated as a SNP.

• 454: A variant locus is a SNP if 1) the majority allele is not a '-' and 2) either a) the number of non-'-' alleles is at least two, or b) there is any non-'-' allele that is not part of a homopolymer run of length ≥ 3.

• Binomial (default): Given an estimated error rate *e *(default 0.005) and sequence alphabet *A*, at a non-polymorphic locus the expected occurrence rate of the second most frequent character (under an equal substitution model) will be *e/*(|*A| - *1). Thus, this method uses a Bonferroni corrected Binomial test to call SNPs: for each locus, we call a SNP if the actual second most frequent character count is greater than *F*^-1^(^1 ^- *α/L*; *d*, *e/*(|*A*| - 1)), where *F *^- 1 ^is the inverse cumulative Binomial distribution function, *α *is the p-value cutoff (default 0.05), *L *is the length of the alignment, and *d *is the read depth at the locus.

• User Provided: The user can provide a file containing a list of loci for each alignment to consider as SNPs, allowing for sophisticated external SNP-callers such as QualitySNP [33] and PyroBayes [32].

### Masking of redundant reads

A read *R_r _*is called "redundant" if the SNPs covered by *R_r _*are a subset of those covered by some other read *R*, and *R_r _*and *R *do not conflict. Hapler scans the reads first in order of first covered SNP position and secondly by length; for each such read *R*, those reads *R_r _*which are made redundant by *R *are subsumed, or "masked" by *R*. *R_r _*is then removed from consideration as a masking read itself. This is necessary to ensure that the compatibility graph $G_{M}^{\prime }$ can be transitively oriented [4]: consider ordered reads *x*, *y *and *z*, where *y *does not share SNPs with *z*. If *x *shares SNPs with both, does not conflict with *y *(which is redundant) but does conflict with *z*, this produces a non-transitive relationship.

### Determination of haplotype blocks and the universal haplotype

For low-diversity data, *G_M _*may consist of multiple connected components. In this case, no information exists to determine whether reads from different connected components could belong to the same haplotype. Thus, for each alignment, haplotypes are assembled for each connected component separately. It can be shown that SNP loci covered by reads of each component form contiguous stretches--hence the usage of the phrase "haplotype block" [34].

Reads in connected components of size one are consistent with all possible haplotypes, and are combined into a "universal" haplotype consistent with all other assembled haplotype regions.

### Determination of block compatibility graphs

Once a connected component *G_M_*_' _has been isolated, the directed, transitive, block compatibility graph $\vec{{G^{\prime }}_{M^{\prime }}}$ is easily created: if read *S_x _*starts before read *S_y _*and does not conflict with *S_y_*, a directed edge is drawn from the node representing *S_x _*in $\vec{{G^{\prime }}_{M^{\prime }}}$ to that representing *S_y_*.

### Minimum coloring

Because we consider only unmasked reads, any compatibility graph $\vec{{G^{\prime }}_{M^{\prime }}}$ is fully transitive: a minimum path cover solves minimum clique cover, solving minimum coloring of the conflict connected component *G_M _*_'_.

**Figure 7:**
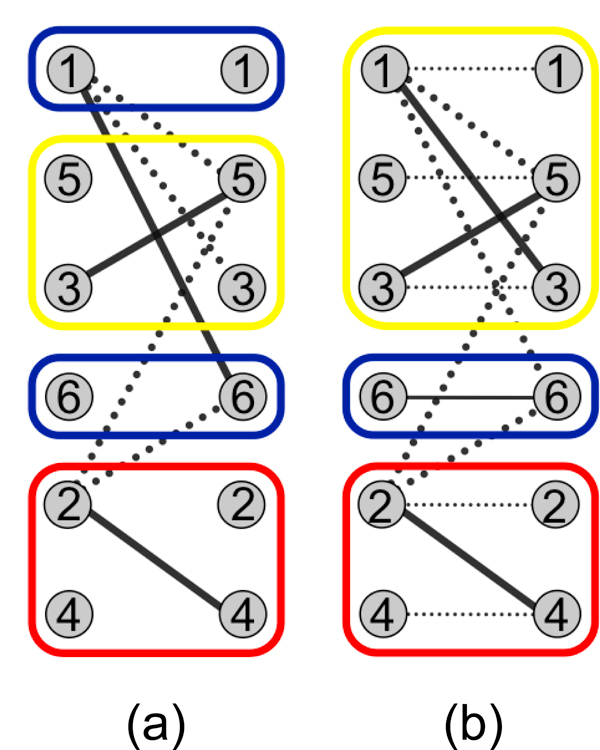
**Unweighted and modified weighted bipartite representations of the conflict graph shown in Figure 1, with associated solutions and colorings shown**. For the unweighted case, multiple minimum colorings are possible: read 1 could be colored blue or yellow. Adding small-weight edges between node pairs maximizes the number of single-node colors. Thus, read 1 is forced to be colored with reads 5 and 3, isolating read 6 which likely contains a sequencing error.

Although the application of Dilworth's theorem in [4] is correct (equating the path covering and coloring numbers), the constructive algorithm that uses a "reachability" bipartite representation is not strictly necessary: any directed acyclic graph can be path covered using a different, "connects-to" bipartite representation [35]. Here, nodes in $\vec{{G^{\prime }}_{M^{\prime }}}$ are represented on the left and right of a bipartite graph *B_M _*_' _, and node *x *from the left of *B_M _*_' _connects to *y *from the right if *x *is directly connected to *y *in $\vec{{G^{\prime }}_{M^{\prime }}}$. Figure 7(a) shows this unweighted representation (and a colored maximum matching) for the conflict graph of Figure 1. This representation affords us two advantages. First, we can modify it to maximize the number of paths (haplotypes) that consist of a single read, isolating reads that are likely to contain errors. This requires the use of a weighted bipartite matching algorithm such as the Hungarian method [36]. Second, we can more effectively take advantage of the mechanics of the Hungarian method to sample in a random fashion from the set of minimum color solutions (see next section).

Regarding the first point, solving a maximum unweighted matching on *B_M _*_' _and computing connected components (after contracting left and right nodes representing the same node in $\vec{{G^{\prime }}_{M^{\prime }}}$) would give a minimum path cover of $\vec{{G^{\prime }}_{M^{\prime }}}$[35]. However, we are given no guarantees about any properties of this path cover, other than its minimality. To remedy this, we modify *B_M _*_' _to produce $B_{M^{\prime }}^{\varepsilon }$ in the following way: Let *n *be the number of nodes in $\vec{{G^{\prime }}_{M^{\prime }}}$. We connect each pair of nodes *x_l _*and *x_r _*(from the left and right of *B_M _*_'_, respectively) representing the same node in $\vec{{G^{\prime }}_{M^{\prime }}}$ by an edge of weight 1 */*(*n *+ 1). Original edges are given weight of 1.

**Theorem 1**. *Computing connected components of a maximum weighted matching of *$B_{M^{\prime }}^{\varepsilon }$*followed by contraction of nodes representing the same node in *$\vec{{G^{\prime }}_{M^{\prime }}}$*produces a minimum path cover of *$\vec{{G^{\prime }}_{M^{\prime }}}$*, while secondarily maximizing the number of paths consisting of a single node*.

*Proof*. As noted in [35], connected components in an unweighted matching of the original *B_M _*_' _produce a minimum path cover of *$\vec{{G^{\prime }}_{M^{\prime }}}$*, where *n *- *k *is the number of paths and *k *is the number of edges selected in the matching. It is easy to see that selection of any edge of weight 1 */*(*n *+ 1) in $B_{M^{\prime }}^{\varepsilon }$ produces a single-node path. It is suffcient to show that the total number of paths produced does not change.

Let  be a maximum unweighted matching of *B_M _*_', _which selects *k *edges. Now consider any maximum weighted matching of $B_{M^{\prime }}^{\varepsilon }$ and let the total weight selected be *k*'. Clearly, *k*' ≥ *k*; *k*' must also be less than *k *+ 1 (otherwise  wasn't a maximum matching). This implies that *k *integer edges and some number of small weight edges are selected. Since *k*' is maximum, the number of single node paths is maximized, while the number of post-contraction edges remains the same as in the unweighted matching: *k*. As before, the number of paths is defined by *n *- *k*.    □

Figure 7(b) shows this modified bipartite graph and the weighted matching produced, which forces the use of the light coloring for read number 1 in Figure 1, isolating the remaining read (number 6) that likely contains a sequencing error.

### Random repetition of coloring and haplotype assembly

In general, colorings of *G_M _*_' _described by Theorem 1 are not unique, and different colorings can produce different haplotypings. If we can sample in a pseudo-random fashion from the coloring solution set a large number of times, we can increase the confidence in our haplotype assemblies: reads that are always similarly colored are likely to be from the same haplotype in reality. This is the final haplotype information used by Hapler. Because the Hungarian method considers augmenting paths based on the order of the inputs, the matching output can be drastically altered for each iteration merely by by randomizing the ordering of nodes in $B_{M^{\prime }}^{\varepsilon }$ before calling the Hungarian method.

Once a set of reads has been identified as belonging to the same haplotype, a haplotype region is assembled. For non-SNP loci covered by reads in the haplotype region, this is the majority vote of the multiple alignment as a whole. For SNP loci, we use the allele of reads belonging to the haplotype. Using the entire alignment in non-SNP positions reduces the possibility of including sequencing errors at non-variant loci, since haplotype-specific coverage is frequently quite low. For loci not covered by reads in the haplotype region, we use a '*~*' character to represent lacking information.

#### Determining assembled haplotype region length

Here we note that haplotype assemblies need not be contiguous, though in practice they almost always are. Consider a multiple alignment of only three reads {*A*, *B*, *C*}, where *A *conflicts with *B*, *B *conflicts with *C*, but *A *and *C *do not overlap. In this case, all minimal colorings will put *A *and *C *together, even though they do not overlap. (Similarly, the universal haplotype will consist of contiguous sequenced regions separated by gaps induced by haplotype blocks.) Thus, for Figures plotting haplotype region lengths (e.g., Figures 2(a), (b), and 3(a)) we actually plot the number of bases in the haplotype region rather than the length. Again, in almost all cases regions are contiguous and this measure is equal to the length, and in the rare situations where this is not the case it underestimates the "length."

### Consensus reconstruction

One possibility for reconstructing an overall consensus sequence is to find a minimum tiling path through haplotype assemblies, from the first base in the alignment to the last. This formulation, however, is complicated by 1) the possibility of haplotype assemblies being non-contiguous (see above) and 2) the existence of the universal haplotype: switching to a universal haplotype region should not be penalized. Thus, we aim to reconstruct the full consensus only at SNP positions, filling in non-SNP loci with the majority vote of the overall alignment. Given a consensus sequence , we define a "possible crossover" as any two adjacent SNPs *S_j _*and *S_k_*, where no haplotype assembly supports (covers and agrees with)  at both *S_j _*and *S_k_*.

A consensus with minimal possible crossovers can easily be computed via dynamic programming. First, for each SNP *S_i _*we identify the set of haplotype assemblies that cover it, call this *H*(*S_i_*). Let *m*(*H_q_*, *S_i_*) be the minimum number of crossovers needed to reconstruct a consensus for the first *i *SNPs (represented in order as *S*_1 _to *S_m _*if there are *m *SNP loci), ending with haplotype assembly *H_q _*providing the allele for *S_i_*. At the base of the dynamic program, for all *H_q _*∈ *H*(*S*_1_), *m*(*H_q_*, *S*_1_) = 0. The solution for other haplotype/SNP combinations can then be computed as

$$\begin{array}{c} \forall H_{q}\in H\left(S_{i}\right),i\in \left\{2...m\right\},m\left(H_{q},S_{i}\right)=\\ \underset{H_{r}\in H\left(S_{i}-1\right)}{\text{min}}\left\{m\left(H_{r},S_{i-1}\right)+1-I_{H_{r}=H_{q}}\right\}. \end{array}$$

In fact, Hapler optimizes the consensus sequence *C *via dynamic programming based on several criteria of decreasing importance:

1. Minimizing the number of possible crossover points in *C*.

2. Maximizing the total supporting read coverage (both redundant and irredundant) of alleles used at SNP positions in *C*.

3. Minimizing the number of unique haplotype assemblies used.

In practice, there may be many paths through haplotype assemblies that minimize possible crossover points. In the case of ties, criterion 2 chooses assemblies for *C *that are high in coverage, minimizing sequencing error and increasing the probability that a possible crossover point is not actually a true crossover point in reality (similar to the uninformed majority vote). Criterion 2 also governs where a necessary crossover from one haplotype assembly to another will occur. If two assemblies overlap at SNP loci, the highest covered will be "crossed into" and "crossed out of" greedily.

**Figure 8:**
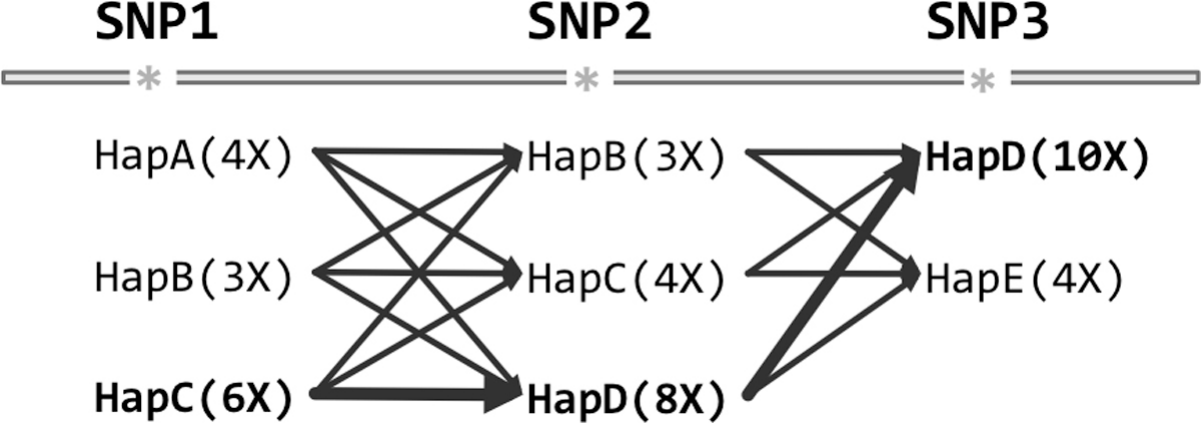
**Dynamic programming representation used for reconstructing minimum chimerism consensus sequences**. At each SNP locus, the haplotype assemblies overlapping that locus are identified, and a minimum chimerism, maximum coverage path is identified. Shown are example haplotype assemblies overlapping three SNPs, as well as the locus-specific coverages of each haplotype assembly.

Criterion 3 will seldom be called upon in practice, as ties in both criteria 1 and 2 are unlikely. Nevertheless, within the constraints of a minimal set of haplotype crossovers maximizing coverage, we prefer to utilize a minimum number of explanatory haplotype regions. A graphical representation can be found in Figure 8; pseudocode can be found in Supplementary File 1.

### Consensus evaluation

Given a set of "true" haplotypes or a set of assembled haplotype regions , Hapler can evaluate the chimerism of a given consensus sequence *C^' ^*against . This is done using a dynamic programming approach similar to that used for consensus reconstruction, with the only differences being 1) SNPs are identified any polymorphic locus in , and 2) *H_C_*(*S_i_*) is used rather than *H*(*S_i_*) where *H_C_*(*S_i_*) defines not only those haplotypes that cover SNP *S_i _*but those that cover and agree with the allele of *C' *at *S_i_*. If no haplotype covers and agrees with *C^' ^*at a SNP *S_i_*, then *S_i _*is associated with a new, unique "error" haplotype for that locus. This will incur two crossovers in the evaluation--one into the unique error haplotype at *S_i _*and one out of it. Thus, errors at SNP loci not supported by any haplotype are heavily penalized. However, sequencing errors in the consensus are not considered if they do not coincide with SNP loci in .

## Competing interests

The authors declare that they have no competing interests.

## Authors' contributions

STO designed the methods, wrote the software, performed the analysis and drafted the manuscript. SJE helped conceive the study, supervised experimental analysis and helped draft the manuscript. Both authors read and approved the final manuscript.

## Supplementary Material

Additional file 1 **An additional PDF file providing Supplementary Materials, including consensus reconstruction pseudocode and supplementary figures (oneil_bmc2011_supp.pdf, Additional File **1**) is available online**. Click here for file

## References

[B1] Pop M Salzberg S Shumway M Genome sequence assembly: algorithms and issues Computer 2002 47 54

[B2] Clark AG Inference of haplotypes from PCR-amplified samples of diploid populations Mol Biol Evol 1990 7 2 111 122 2108305 10.1093/oxfordjournals.molbev.a040591

[B3] Salem R Wessel J Schork N A comprehensive literature review of haplotyping software and methods for use with unrelated individuals Human Genomics 2005 2 39 66 15814067 10.1186/1479-7364-2-1-39 PMC3525117

[B4] Eriksson N Pachter L Mitsuya Y Rhee S Wang C Gharizadeh B Ronaghi M Shafer R Beerenwinkel N Viral Population Estimation Using Pyrosequencing PLoS Computational Biology 2008 4 5 e1000074+ 18437230 10.1371/journal.pcbi.1000074 PMC2323617

[B5] Zagordi O Klein R DÃumer M Beerenwinkel N Error correction of next-generation sequencing data and reliable estimation of HIV quasispecies Nucleic Acids Research 2010 38 21 7400 7409 10.1093/nar/gkq655 20671025 PMC2995073

[B6] Wheat C Rapidly developing functional genomics in ecological model systems via 454 transcriptome sequencing Genetica 2010 138 4 433 451 10.1007/s10709-008-9326-y 18931921

[B7] Turner T Hahn M Locus- and population-specific selection and differentiation between incipient species of *Anopheles gambiae* Molecular Biology and Evolution 2007 24 9 2132 2138 10.1093/molbev/msm143 17636041

[B8] Vera C Wheat C Fescemyer H Frilander M Crawford D Hanski I Marden J Rapid transcriptome characterization for a nonmodel organism using 454 pyrosequencing Molecular Ecology 2008 17 7 1636 1647 10.1111/j.1365-294X.2008.03666.x 18266620

[B9] Anderson JT Mitchell-Olds T Ecological genetics and genomics of plant defences: evidence and approaches Funct Ecol 2011 25 312 324 10.1111/j.1365-2435.2010.01785.x 21532968 PMC3082142

[B10] O'Neil ST Dzurisin JDK Carmichael R Lobo NF Emrich SJ Hellmann JJ Population level transcriptome sequencing of nonmodel organisms *Erynnis propertius *and *Papilio zelicaon* BMC Genomics 2010 11 310+ 10.1186/1471-2164-11-310 20478048 PMC2887415

[B11] Ewen-Campen B Shaner N Panfilio KA Suzuki Y Roth S Extavour CG The maternal and early embryonic transcriptome of the milkweed bug *Oncopeltus fasciatus* BMC Genomics 2011 12 61 10.1186/1471-2164-12-61 21266083 PMC3040728

[B12] O'Neil ST Emrich SJ Robust haplotype reconstruction of eukaryotic read data with Hapler ICCABS '11: Proceedings of the IEEE 1st International Conference on Computational Advances in Bio and medical Sciences 141 146

[B13] Lancia G Bafna V Istrail S Lippert R Schwartz R SNPs problems, complexity, and algorithms ESA '01: Proceedings of the 9th Annual European Symposium on Algorithms 2001 182 193

[B14] Bonizzoni P Vedova GD Dondi R Li J The haplotyping problem: an overview of computational models and solutions Journal of Computer Science and Technology 2003 18 6 675 688 10.1007/BF02945456

[B15] Schwartz R Theory and algorithms for the haplotype assembly problem Communications in Information and Systems 2010 10 23 38

[B16] Xie M Wang J Chen J Wu J Liu X Computational models and algorithms for the single individual haplotyping problem Current Bioinformatics 2010 5 18 28 10.2174/157489310790596411

[B17] Bafna V Istrail S Lancia G Rizzi R Polynomial and APX-hard cases of the individual haplotyping problem Theoretical Computer Science 2005 335 109 125 10.1016/j.tcs.2004.12.017

[B18] Cilibrasi R Leo V Kelk S Tromp J The complexity of the single individual SNP haplotyping problem Algorithmica 2007 49 13 36 10.1007/s00453-007-0029-z

[B19] Li Z Wu L Zhao Y Zhang X A dynamic programming algorithm for the k-haplotyping problem Acta Mathematicae Applicatae Sinica 2006 22 3 405 412 10.1007/s10255-006-0315-6

[B20] Panconesi A Sozio M Fast hare: a fast heuristic for single individual SNP haplotype reconstruction Algorithms in Bioinformatics 2004 266 277

[B21] He D Choi A Pipatsrisawat K Darwiche A Eskin E Optimal algorithms for haplotype assembly from whole-genome sequence data Bioinformatics 2010 26 12 i183 10.1093/bioinformatics/btq215 20529904 PMC2881399

[B22] Astrovskaya I Tork B Mangul S Westbrooks K Mandoiu I Balfe P Zelikovsky A Inferring viral quasispecies spectra from 454 pyrosequencing reads BMC Bioinformatics 2011 12 Suppl 6 S1 10.1186/1471-2105-12-S6-S1 21989211 PMC3194189

[B23] Denisov G Walenz B Halpern AL Miller J Axelrod N Levy S Sutton G Consensus generation and variant detection by Celera Assembler Bioinformatics 2008 24 8 1035 1040 10.1093/bioinformatics/btn074 18321888

[B24] Dincă V Zakharov E Hebert P Vila R Complete DNA barcode reference library for a country's butterfly fauna reveals high performance for temperate Europe Proceedings of the Royal Society B: Biological Sciences 2011 278 1704 347 10.1098/rspb.2010.1089 PMC3013404 20702462

[B25] Zakharov E Hellmann J Genetic differentiation across a latitudinal gradient in two co-occurring butterfly species: revealing population differences in a context of climate change Molecular Ecology 2008 17 189 208 10.1111/j.1365-294X.2007.03488.x 17784923

[B26] Lawniczak M Emrich S Holloway A Regier A Olson M White B Redmond S Fulton L Appelbaum E Godfrey J Widespread divergence between incipient *Anopheles gambiae *species revealed by whole genome sequences Science 2010 330 6003 512 10.1126/science.1195755 20966253 PMC3674514

[B27] Lee S Cheran E Brudno M A robust framework for detecting structural variations in a genome Bioinformatics 2008 24 13 i59 10.1093/bioinformatics/btn176 18586745 PMC2718654

[B28] Davidson S Research suggests importance of haplotypes over SNPs Nature Biotechnology 2000 18 11 1134 1135 10.1038/81100 11062421

[B29] Hoehe M Timmermann B Lehrach H Human inter-individual DNA sequence variation in candidate genes, drug targets, the importance of haplotypes and pharmacogenomics Current Pharmaceutical Biotechnology 2003 4 351 378 10.2174/1389201033377300 14683431

[B30] Pop M Kosack D Using the TIGR assembler in shotgun sequencing projects Methods in Molecular Biology 2004 255 279 294 15020832 10.1385/1-59259-752-1:279

[B31] Li H Handsaker B Wysoker A Fennell T Ruan J Homer N Marth G Abecasis G Durbin R The sequence alignment/map format and SAMtools Bioinformatics 2009 25 16 2078 10.1093/bioinformatics/btp352 19505943 PMC2723002

[B32] Quinlan AR Stewart DA Stromberg MP Marth GT Pyrobayes: an improved base caller for SNP discovery in pyrosequences Nature Methods 2008 5 2 179 181 10.1038/nmeth.1172 18193056

[B33] Tang J Vosman B Voorrips R Van Der Linden C Leunissen J QualitySNP: a pipeline for detecting single nucleotide polymorphisms and insertions/deletions in EST data from diploid and polyploid species BMC Bioinformatics 2006 7 438 10.1186/1471-2105-7-438 17029635 PMC1618865

[B34] Wall J Pritchard J Haplotype blocks and linkage disequilibrium in the human genome Nature Reviews Genetics 2003 4 8 587 597 10.1038/nrg1123 12897771

[B35] Nemhauser G Trotter L Jr Nauss R Set partitioning and chain decomposition Management Science 1974 20 11 1413 1423 10.1287/mnsc.20.11.1413

[B36] Kuhn H The Hungarian method for the assignment problem Naval Research Logistics Quarterly 1955 2 1-2 83 97 10.1002/nav.3800020109

