# Supplementary material for: Haplotype and minimum-chimerism consensus determination using short sequence data
Source: BMC Genomics. 2012 Apr 12;13(Suppl 2):S4. doi: 10.1186/1471-2164-13-S2-S4 (PMC3394418; doi:10.1186/1471-2164-13-S2-S4)
Supplement: Additional file 1 — An additional PDF file providing Supplementary Materials, including consensus reconstruction pseudocode and supplementary figures (oneil_bmc2011_supp.pdf, Additional File 1) is available online. [file 1471-2164-13-S2-S4-S1.pdf]

Supplementary Materials

Shawn T. O'Neil[1] , Scott J. Emrich*[1]

[1]Department of Computer Science and Engineering, University of Notre Dame, Notre Dame, IN 46556, USA

Email: STO: soneil@nd.edu; SJE*: semrich@nd.edu;

*Corresponding author

Consensus reconstruction pseudocode

Algorithm 1 is part of the dynamic programming solution for computing a minimum chimerism consensus given a set of assembled haplotype regions covering a set of SNP loci. SNPs are sorted by position and are indexed from S_1 to S_m. The function $d(H_k, S_i)$ gives the number of reads in haplotype H_k that are covering the locus of S_i; $H(S_i)$ is the set of haplotypes that cover SNP S_i. The variable $m(H_k, S_i)$ records the minimum cost of reconstructing a consensus from S_1 to S_i ending with H_k providing the allele for S_i. This cost is represented as a triple, recording number of chimerisms (to be minimized), SNP allele coverage of the consensus (to be maximized), and unique haplotypes used (to be minimized): $\langle MinChim, MaxCov, Usage \rangle$. When comparing two triples, first the $MinChim$ dimension is considered, in the case of ties the $MaxCov$ dimension is considered, and in the case of further ties the number of elements in the $Usage$ dimension are considered. Ties in all three dimensions are broken arbitrarily.
After algorithm 1 is run, the minimum over H_k of $m(H_k, S_m)$ is found, and backpointers are traced back to determine the final consensus sequence.

Algorithm 1: Minimum Chimerism Consensus Reconstruction

- **foreach** $H_i \in H(S_1)$ **do**
  - $m(H_i, S_1) = \langle 0, d(H_i, S_1), \{H_i\} \rangle;$
  - $backPointer(H_i, S_1) = null;$
- **foreach** $S_i \in S_2 \dots S_m$ **do**
  - **foreach** $H_k \in H(S_i)$ **do**
    - $bestFound = \langle \infty, -1, null \rangle;$
    - $bestFoundFrom = null;$
    - **foreach** $H_j \in H(S_{i-1})$ **do**
      - $\langle jMinChim, jMaxCov, jUsage \rangle = m(H_j, S_{i-1});$
      - **if** $H_j == H_k$ **then**
        - $pkMinChim = jMinChim;$
        - $pkMaxCov = jMaxCov + d(H_k, S_i);$
        - $pkUsage = jUsage;$
      - **else**
        - $pkMinChim = jMinChim + 1;$
        - $pkMaxCov = jMaxCov + d(H_k, S_i);$
        - $pkUsage = jUsage \cup \{H_k\};$
      - **if** $\langle pkMinChim, pkMaxCov, pkUsage \rangle \prec bestFound$ **then**
        - $bestFound = \langle pkMinChim, pkMaxCov, pkUsage \rangle;$
        - $bestFoundFrom = \langle H_j, S_{i-1} \rangle;$
    - $m(H_k, S_i) = bestFound;$
    - $backPointer(H_k, S_i) = bestFoundFrom;$

Supplementary figures

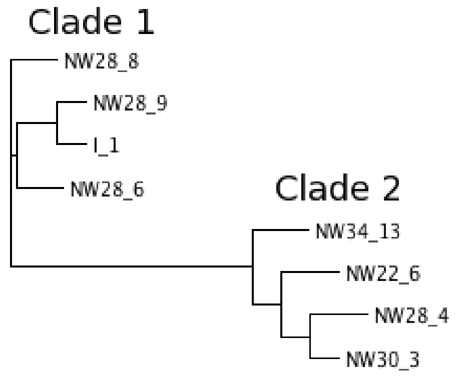

Figure 1: Cladogram showing relative genetic divergence between haplotype sequences included in Clade 1 (which constitutes the low diversity test dataset) and Clade 2 (which, when combined with Clade 1, represents the high diversity test dataset).

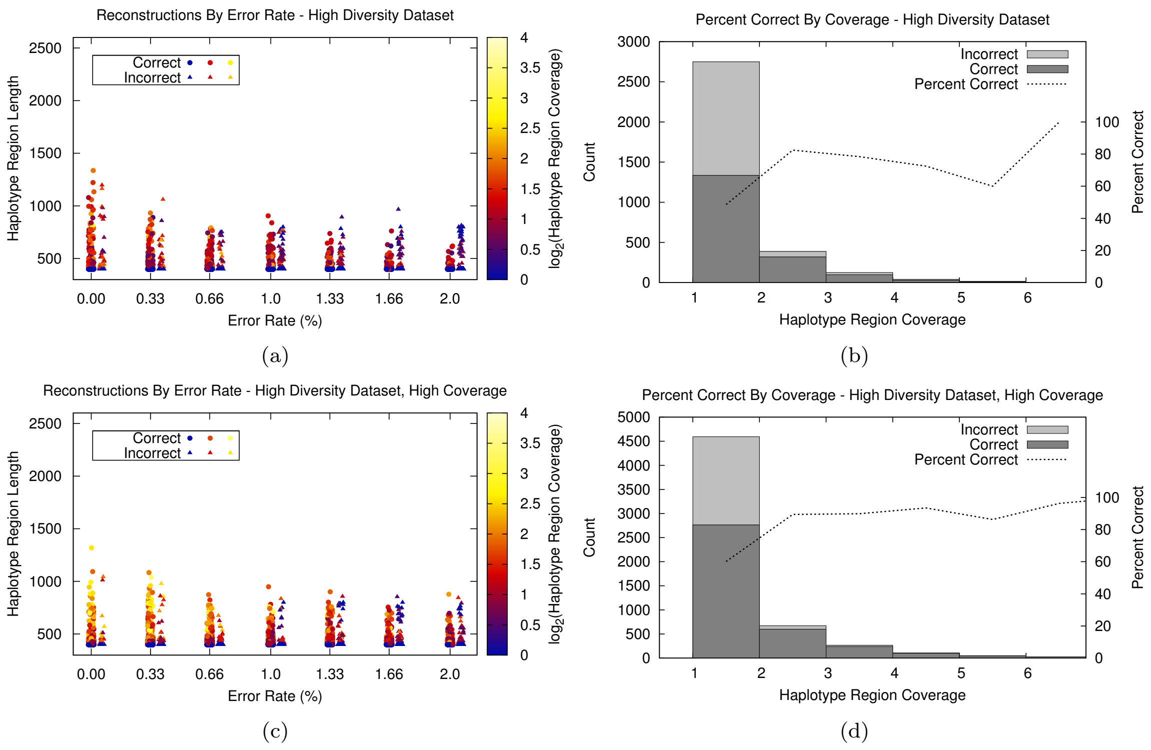

Figure 2: Haplotype assemblies varying error rate for the high diversity dataset over five trials of sequencing to 24X total coverage (3X per haplotype). Supplementary figure 2(b) aggregates all results of 2(a) binned by coverage, identifying counts and percents of correct versus incorrect assemblies. For supplementary figures 2(c) and 2(d), coverage is doubled to 6X per haplotype, improving the length and correctness of haplotype assemblies.

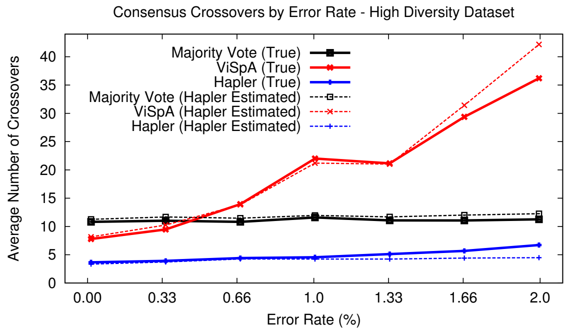

Figure 3: Chimerism analysis varying error rate for Hapler, majority vote, and ViSpA consensus sequences for the high diversity dataset at 3X per haplotype sequencing coverage. True crossover numbers indicate the minimum number of crossovers through sequenced haplotypes needed to reconstruct the consensus. Hapler estimated crossover numbers indicate the minimum number of crossovers through Hapler-assembled haplotype regions needed to reconstruct the consensus. Each datapoint represents an average of 50 measurements.

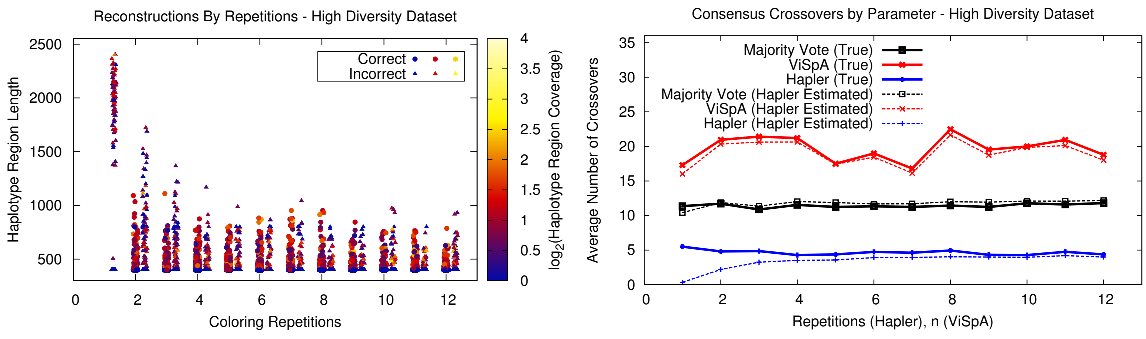

(a) (b)

Figure 4: Haplotype assemblies and consensus chimerism analysis varying coloring repetitions (for Hapler) and n (for ViSpA) for the high diversity dataset. As with the low diversity results, low repetition numbers result in long but chimeric haplotype assemblies, while reconstructed consensus sequences show low true chimerism even when reconstructed from chimeric assemblies.
